# Budesonide, fluticasone propionate, and azithromycin do not modulate the membrane vesicle release by THP-1 macrophages and respiratory pathogens during macrophage infection

**DOI:** 10.1007/s10787-017-0359-7

**Published:** 2017-05-20

**Authors:** Charlotte Volgers, Gert E. Grauls, Pauline H. M. Hellebrand, Paul H. M. Savelkoul, Frank R. M. Stassen

**Affiliations:** 1 Department of Medical Microbiology, School of Nutrition and Translational Research in Metabolism (NUTRIM), Maastricht University Medical Centre, P. Debyelaan 25, 6229 HZ Maastricht, The Netherlands; 2 Department of Medical Microbiology and Infection Control, VU University Medical Center, Amsterdam, The Netherlands

**Keywords:** Membrane vesicles, Bacterial infection, Budesonide, Fluticasone propionate, Azithromycin, Non-typeable *Haemophilus influenzae*, *Moraxella catarrhalis*, *Streptococcus pneumoniae*, *Pseudomonas aeruginosa*

## Abstract

Patients with more severe chronic obstructive pulmonary disease frequently experience exacerbations and it is estimated that up to 50% of these exacerbations are associated with bacterial infections. The mainstay treatment for these infection-related exacerbations constitutes the administration of glucocorticoids, alone or in combination with antibiotics. A recent line of evidence demonstrates that many hormones including the steroid beclomethasone can also directly affect bacterial growth, virulence, and antibiotic resistance. The effect of these regimens on the release of potentially virulent and toxic membrane vesicles (MVs) is at present unclear. In this study, we determined the effect of several pharmacological agents on MVs release by and bacterial growth of common respiratory pathogens. We found that neither the release of MVs nor the bacterial growth was affected by the glucocorticoids budesonide and fluticasone. The macrolide antibiotic azithromycin only inhibited the growth of *Moraxella catarrhalis* but no effects were observed on bacterial MV release at a concentration that is achieved locally in the epithelial lining on administration. The macrophage pro-inflammatory response to MVs was significantly reduced after treatment with budesonide and fluticasone but not by azithromycin treatment. Our findings suggest that these glucocorticoids may have a positive effect on infection-related inflammation although the bacterial growth and MV release remained unaffected.

## Introduction

Chronic obstructive pulmonary disease (COPD) is a progressive airway disease that is characterized by excessive inflammation resulting in airway limitation and a progressive decline in lung function (Barnes 2008). Moreover, patients with moderate and severe disease often experience exacerbations of disease (O’Reilly et al. 2006). These exacerbations are frequently triggered by infectious insults and it has been estimated that bacterial infections account for up to 50% of the exacerbations (Sethi and Murphy 2008).

Standard treatment of patients that experience an exacerbation consists of the administration of systemic corticoids alone or combined with antibiotics (Walters et al. 2009; Laue et al. 2015; Ram et al. 2016). Moreover, it has been shown in several large randomized trials that patients with stable disease may benefit from the use of inhaled corticosteroids (ICS) and antibiotics as these can reduce the number of exacerbations (Donath et al. 2013; Han et al. 2014; Kew et al. 2014). Apart from their antibacterial effects, macrolide antibiotics also have been shown to reduce inflammation (Čulić et al. 2001; Brusselle and Joos 2014). ICS, on the other hand, may have controversial effects on the defence against bacteria. Although they are able to reduce epithelial invasion by airway pathogens, this might be at the expense of resistance to infections as ICS also inhibit the release of antimicrobial peptides and suppress inflammatory processes (Mitchell et al. 2007; Barbier et al. 2008; Wang et al. 2013). Alternatively, ICS may also have a direct effect on the bacteria as recent studies have shown that hormones such as catecholamines and steroid hormones can also directly affect bacterial growth, virulence, and gene expression (Freestone et al. 2013; Earl et al. 2015).

Bacterial membrane vesicles (MVs) are released by bacteria in response to a variety of stressors. These nanosized (30–300 nm) MVs do not only contribute to bacterial virulence, but also increase the resistance to certain antimicrobial peptides and antibiotics. Moreover, they can exert strong pro-inflammatory responses (Schwechheimer et al. 2014; Kaparakis-Liaskos and Ferrero 2015). Therefore, understanding bacterial behavior in terms vesiculation may help to improve treatment strategies. Moreover, host cells are also known to release MVs (Koifman et al. 2017), and several pro-inflammatory properties have been assigned to MVs released in the context of infection and inflammation (Yáñez-Mó et al. 2015; Schorey and Harding 2016).

In this study, we aimed to address if treatment with the glucocorticoids budesonide and fluticasone and the antibiotic azithromycin (a) affects MV release by bacteria and macrophages, and (b) suppresses the pro-inflammatory response to these MVs released by several common respiratory bacterial pathogens.

## Materials and methods

### Reagents and antibodies

Budesonide (BUD), fluticasone propionate (FLUT), and azithromycin (AZI) were from Sigma (Sigma Aldrich, St. Louis, MO, USA). The anti-*Haemophilus influenzae* type b (α-Hib; clone 1079/457) monoclonal antibody was obtained from Acris (Acris GmbH, Herford, Germany). The rabbit serum against *Moraxella catarrhalis* (Mrc, strain A 1.39 N, isolated from children in a primary school in Nieuwegein, the Netherlands, 1989) was kindly provided by Dr. J. Hays (Erasmus University, Rotterdam, the Netherlands). The polyclonal antibody against *Pseudomonas aeruginosa* (Psa) (OAMA02609) was from Antibodies online (Aviva Systems Biology, San Diego, CA, USA). α-CD63 (unconjugated, mouse-anti-human clone H5C6) and α-CD81 (PE-conjugated, mouse-anti-human clone JS-81) were from BD (BD Bioscience, Franklin Lakes, NJ, USA). Purification of antibodies from serum was performed using the antibody serum purification kit based on protein A (Abcam, Cambridge, MA, USA). Antibodies for detection for flow cytometric analyses were PE-conjugated using a PE-labeling kit from Abcam according to the manufacturers’ instructions (Cambridge, MA, USA).

### Bacterial strains and culture

The following bacterial strains were selected: *Haemophilus influenzae* (NTHi, ATCC-49247), *Pseudomonas aeruginosa* (Psa, ATCC-27853), *Streptococcus pneumoniae* (Spn, ATCC-49619), and a clinical *Moraxella catarrhalis* (Mrc) isolate (University Medical Centre Maastricht (MUMC+), the Netherlands). The ATCC strains are well characterized and recommended by ATCC for quality control and antimicrobial susceptibility testing. All bacteria were cultured overnight on blood plates except for NTHi which was cultured on vitalex-supplemented chocolate agar plates (Oxoid, Wesel, Germany) in 5% CO_2_ at 37 °C. After overnight pre-culture, bacteria were resuspended at 0.5 McFarland (1.5 × 10^8^ colony forming units (cfu)/ml) in RPMI1640 and used for infection or culture experiments. For bacterial culture, bacteria were used at 5 × 10^7^ cfu/ml and culture without or with BUD, FLUT, or AZI for 6 h in 10 ml RPMI1640. Next, the conditioned media were processed by centrifugation at 1200×*g* for 10 min, at room temperature. The pelleted bacteria were washed, diluted in PBS, and the optical density was determined at 600 nm using optical methacrylate disposable cuvettes (Sarstedt, Newton, NC, USA). The supernatants were centrifuged again at 1200×*g* for 10 min, and the supernatants were filtered through 0.22 µM filters. Hereafter, the supernatants were further concentrated 20 times to a total of 500 µl by centrifugation at 4000×*g* for 15 min using Amicon Ultra-15 10-kDa centrifugal filter units (Millipore, Billerica, MA, USA).

MVs used for the stimulation of THP1 macrophages were obtained from bacterial cultures (at a density of 1 × 10^8^ cfu/ml) following culturing for 4 h in 30 ml complete vesicle-depleted medium containing 5% FCS, that was obtained as described in the “Cells and media” section. Upon culture, supernatants were depleted from bacteria by two centrifugation steps at 1200×*g* for 10 min and 0.22 µm filtration. The supernatants that were cleared from bacteria were then further processed by ultrafiltration and size-exclusion chromatography (SEC), as described below.

### Cells and media

The human monocytic cell line THP-1 (ATCC-TIB202) was maintained in RPMI1640 (Sigma, St. Louis, MO, USA) supplemented with 100 mM sodium pyruvate, 22.5% glucose, 25 mM β-mercaptoethanol, and 10% fetal calf serum (FCS) (Lonza, Verviers, Belgium) and cultured in 5% CO_2_ at 37 °C. For monocyte differentiation, cells were seeded in a 24-well plate at 0.5 × 10^6^ cells/well or in a 96-well plate at 1 × 10^4^ cells/well and stimulated for 72 h with 200 nM phorbol 12-myristate 13-acetate (PMA; Sigma, St. Louis, MO, USA). THP-1 macrophage stimulations were performed in vesicle-depleted medium containing 5% FCS (complete vesicle-depleted medium). This medium was obtained by combining vesicle-depleted RPMI1640 medium with 30% FCS with FCS-free medium (both supplemented with sodium pyruvate and glucose). Vesicle-depleted medium was generated by overnight centrifugation at 100,000×*g* using a 70Ti-rotor, κ-factor 44 in an Optima L-90 K ultracentrifuge (both Beckman Coulter, Fullerton, CA, USA).

### Macrophage infection for membrane vesicle analysis

THP-1 macrophages seeded in 24-well plates were washed three times with PBS, and medium was replaced with complete vesicle-depleted medium. Hereafter, the cells were pre-treated with BUD (0.1 µM), FLUT (0.1 µM), or AZI (3 µg/ml) for 1 h, and the concentrations were previously calculated to represent a concentration as can be obtained locally upon administration by inhalation (Wagner et al. 2015; Olsen et al. 1996; Ek et al. 1999). After pretreatment, macrophages were infected with one of the bacteria at a multiplicity of infection of ten for 6 h. After infection, the medium was harvested, processed by centrifugation at 300×*g* and 1200×*g*, filtrated with a 0.22-µM micropore filter, and subsequently analyzed by flow cytometry.

### MV concentration and purification from conditioned medium using ultrafiltration and SEC

The bacteria-cleared conditioned media obtained from bacterial cultures were subjected to ultrafiltration (Lobb et al. 2015) and SEC (Böing et al. 2014). First, the cleared conditioned media were concentrated to 500 µl in 2 runs at 4000×*g* for 15 min at room temperature using Amicon Ultra-15 10-kDa centrifugal filter units (Millipore, Billerica, MA, USA) (Lobb et al. 2015). Then, the concentrates were purified by SEC using Sepharose columns as described by Boïng et al. with minor modifications (Böing et al. 2014). For this, a 15-ml TELOS filtration column (Kinesis Scientific Experts, St. Neots, Cambridgeshire, UK) was stacked with a total of 10 ml Sepharose CL-2B (GE Healthcare, Uppsala, Sweden). The concentrates were loaded onto the column and fractions of 0.5 ml were eluted using PBS. The fractions that were found to be highly enriched for MVs and negative for free protein (fractions 7–11 as were determined by flow cytometry and Micro BCA, Pierce, Rockford, IL, USA) were pooled and stored at −80 °C until further use.

### Flow cytometric analysis of MVs using antibody-coated latex beads

A bead-based flow cytometric assay based on a method that was previously described by Volgers et al. (2017) was used for the semi-quantitative analysis of bacterial MVs and CD63^+^/CD81^+^ host cell-derived MVs. Four-micrometer-sized aldehyde–sulfate beads were washed in MES buffer, coated with α-CD63 antibody, or antibodies directed against bacterial vesicles (with the exception of Spn as we could not establish this assay for Spn). Antibody-coated beads were incubated overnight with processed supernatants (200 µl) obtained after infection under constant agitation at 1000 rpm at room temperature. Next, the beads were washed twice with PBS filtered through a 0.22-µM filter containing 2% (w/v) bovine serum albumin (BSA) and incubated with PE-conjugated detection antibodies (α-CD81-PE for host cell vesicles or an PE-conjugated α-bacterial antibody) for 90 min under continuous agitation at room temperature. Bacterial antibodies were PE-conjugated using a PE-conjugation kit (Abcam, Cambridge, MA, USA) according to the manufacturer’s instructions. Then, the vesicle-bead complexes were washed, suspended in PBS (300 µl), and used for flow cytometric analysis using a FACSCanto™ (BD Bioscience, Franklin Lakes, NJ, USA). The lower threshold for detection was set at 2% based on the percentage of PE-positive control beads (based on culture medium). Analyses were performed using FACSDiva Software. The relative amount of MVs was calculated by the multiplication of the percentage of positive beads with the median fluorescence intensity and expressed relative to the control (as % of control).

### MV analysis by tunable resistive pulse sensing

Tunable resistive pulse sensing analysis was used to determine the MV-concentration using the qNano Gold, the Izon Control Suite Software v3.2, and the reagent kit (type RK1) for EV analysis from Izon (Izon Science Ltd., Oxford, UK). The measurements were performed using a NP150-pore. The stretch was fixed at 47 mm, the pressure kept at 6 mbar, and a baseline current of ±100 nA was maintained. Solution G was added (10%) to supernatants that were diluted in solution Q (1:1), and each sample was measured for 10 min and measurements were repeated when system instabilities occurred. The samples were calibrated using 114-nm polystyrene calibration beads (CPC100, Izon Science Ltd., Oxford, UK) at a concentration of 1 × 10^9^ particles/ml diluted in culture medium.

### Macrophage stimulation with MVs 

Stimulation of THP-1 macrophages with MVs was performed using macrophages seeded in 96-well plates. Prior to stimulation, the cells were washed three times with PBS whereafter the medium was replaced with complete vesicle-depleted medium. Then the cells were pre-treated with BUD (0.1 µM), FLUT (0.1 µM), or AZI (3 µg/ml) for 1 h. Next, the cells were washed again after which they were exposed to 20 µl of the MV-containing SEC fractions, without or in the presence of BUD, FLUT, or AZI, in complete vesicle-depleted medium for 16 h. Hereafter, the culture supernatants were harvested and used for cytokine measurements.

### Cytokine measurements

TNF-α levels in the supernatants from the stimulation experiments were determined by enzyme-linked immunosorbent assay (ELISA) using the human Ready-Set-Go TNF-α ELISA kit eBioscience (Affymetrix eBioscience, Santa Clara, CA, USA).

### Statistical analysis

Statistical analysis was performed using GraphPad Prism 5 Software (GraphPad, San Diego, CA, USA). Statistical dispersion was determined by calculating the standard error of the mean. A Mann–Whitney *t* test was performed for the statistical analysis of the variance between the means of two groups. *P* values were considered significant when <0.05.

## Results

### Effect of BUD, FLUT, and AZI on bacterial and host cell membrane vesicle release during infection

**Fig. 1:**
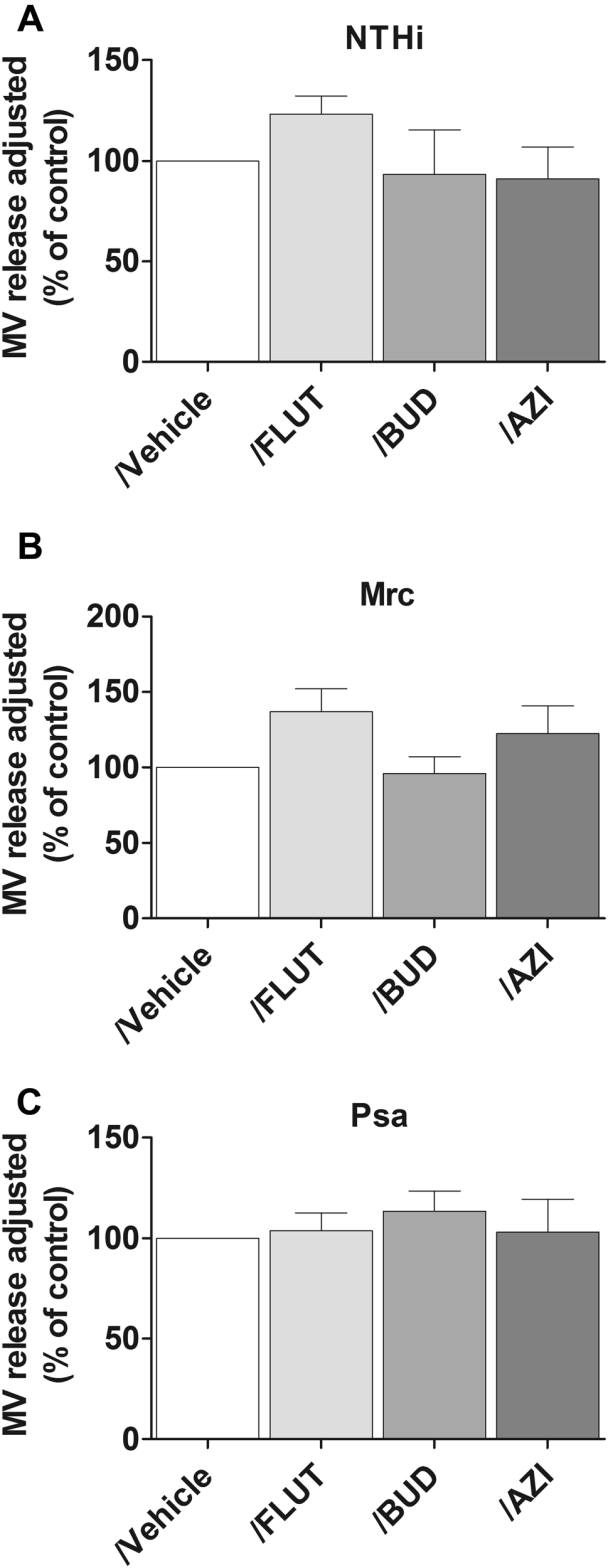
Flow cytometric analysis of the bacterial MV release during macrophage infection in the presence or absence of pharmacological agents. Macrophages were infected with NTHi (**a**), Mrc (**b**), or Psa (**c**) for 4 h at an MOI of 10 in the continuous presence of FLUT, BUD, or AZI. The relative counts are expressed here as the percentage of the untreated control condition (*n* = 3)

First, the effect of BUD, FLUT, and AZI on bacterial MV release was determined. This was done semi-quantitatively by flow cytometry based on antibody-coated beads. We found that neither FLUT nor BUD significantly affected the vesicle release by NTHi and Mrc (Fig. 1a, b). Treatment with AZI also did not affect the MV release (Fig. 1c).

**Fig. 2:**
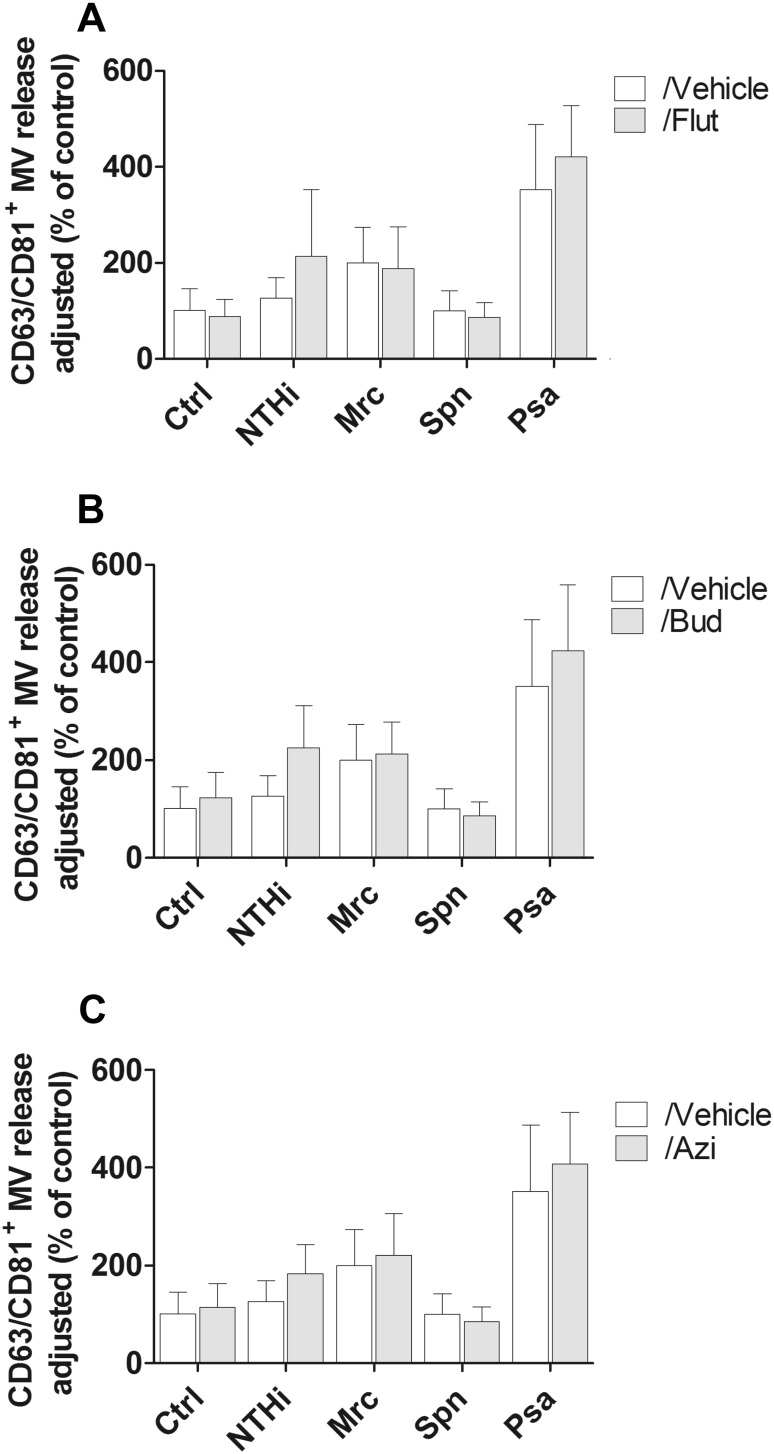
Flow cytometric analysis of CD63^+^/CD81^+^ MV release by macrophages during infection in the presence or absence of pharmacological agents. Macrophages were infected with NTHi, Mrc, Spn, or Psa for 4 h at an MOI of 10 in the continuous presence of FLUT (**a**), BUD (**b**), or AZI (**c**). Data are expressed here as the percentage of the untreated control condition (*n* = 3)

The release of MVs is highly conserved and apart from the bacteria the host cells are also known to release membrane vesicles. We therefore assessed the effect of BUD, FLUT, and AZI treatment on the release of CD63^+^/CD81^+^ host cell vesicles by macrophages under control conditions and upon infection. Neither treatment with BUD and FLUT, nor that with AZI, was found to affect the release of host cell MVs (Fig. 2).

### Bacterial MV release in response to BUD, FLUT, and AZI

**Fig. 3:**
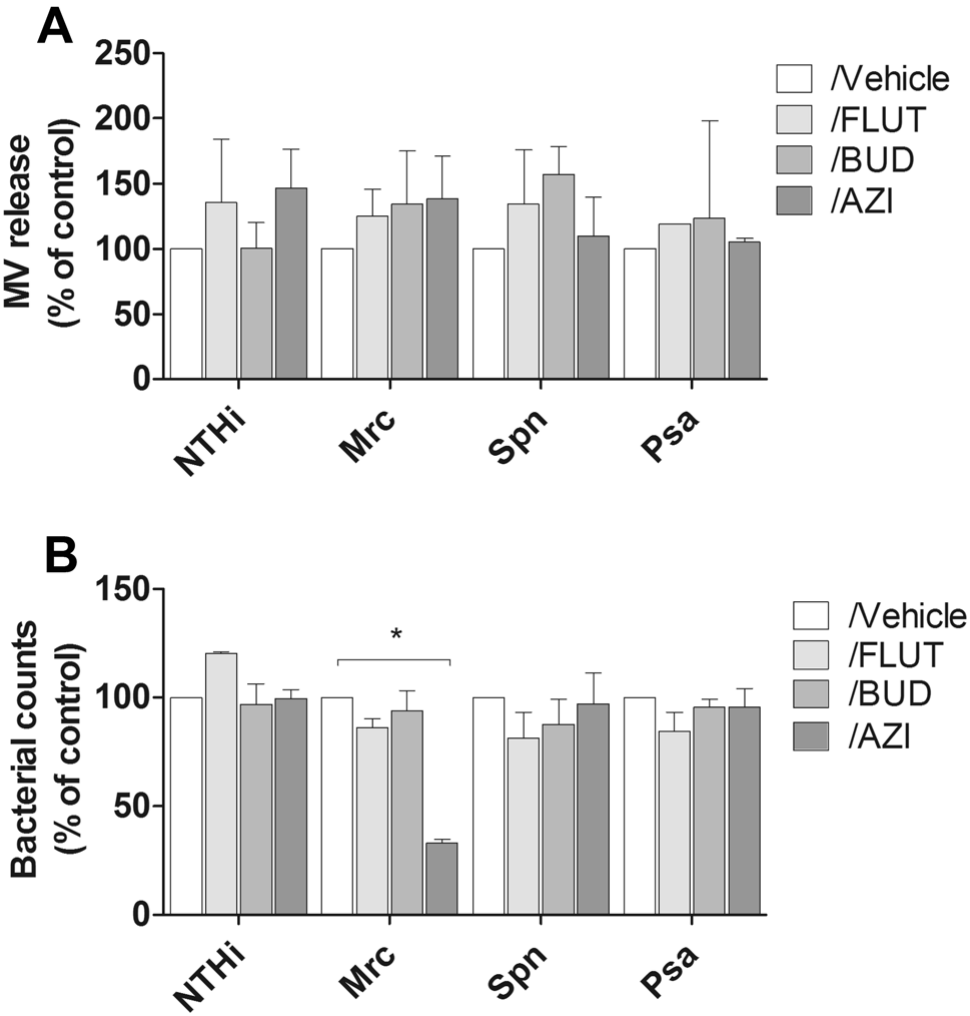
Effect of BUD, FLUT, and AZI on the MV release and bacterial growth during culture. NTHi, Mrc, Spn, or Psa were cultured for 6 h in the continuous presence of FLUT, BUD, or AZI. MV release was determined by TRPS analysis (**a**) and the bacterial growth was determined with the optical density of the bacterial suspensions at 600 nm (**b**) (*n* = 3, *P < 0.05)

Next, we aimed to determine how the bacterial MV release is affected by BUD, FLUT, and AZI in the absence of host cells. Vesicle release after 6 h of culture without or with these drugs was determined by tunable resistive pulse sensing analysis. We observed that treatment with neither FLUT, BUD, nor AZI affected the release of bacterial MVs (Fig. 3a).

Next, we determined if FLUT, BUD, or AZI had any effect on the bacterial growth. To assess this, the relative number of bacteria was determined upon treatment with FLUT, BUD, and AZI, which was done by measuring the optical density. As shown in Fig. 3b, only the Mrc counts were significantly reduced after bacterial culture in the presence of AZI.

### Effect of BUD, FLUT, and AZI on the TNF-α response to immuno-stimulatory bacterial membrane vesicles

**Fig. 4:**
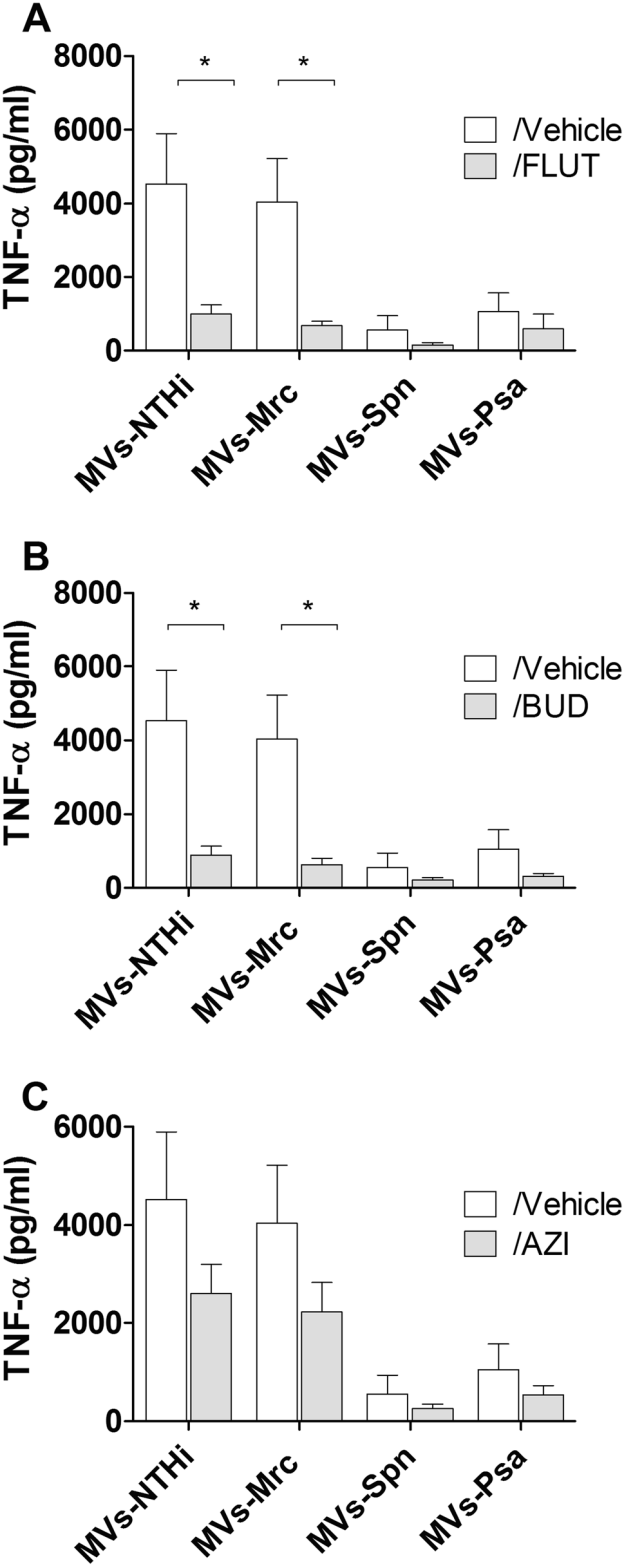
Effect of FLUT, BUD, and AZI on the TNF-α release by naïve macrophages in response to bacteria MVs. Macrophages were pre-treated for 1 h with FLUT (**a**), BUD (**b**), or AZI (**c**), before they were exposed overnight to purified MVs released during culture of NTHi, Mrc, Spn, or Psa. The TNF-α release was determined by ELISA (*n* = 4, *P < 0.05)

Since bacterial MVs can possess a strong pro-inflammatory character (Macdonald 2013; Ellis and Kuehn 2010; Schaar et al. 2011a; Ren et al. 2012; Olaya-Abril et al. 2014), our next aim was to establish if BUD, FLUT, and AZI affected the TNF-α release by naïve macrophages in response to bacteria membrane vesicles. MVs from both NTHi and Mrc induced the release of TNF-α by macrophages, while the response to MVs from Psa and Spn was less pronounced. Treatment with BUD and FLUT significantly reduced the release of TNF-α by macrophages in response to bacterial MVs from NTHi and Mrc (Fig. 4a, b). In contrast, the TNF-α release in response to Spn and Psa MVs was not affected by BUD or FLUT. No significant effect of AZI was found on the TNF-α response to either NTHi-, Mrc-, Spn-, or Psa-derived MVs (Fig. 4c).

## Discussion

In this study, we determined if the glucocorticoids budesonide (BUD) and fluticasone propionate (FLUT), and the antibiotic azithromycin (AZI) affected the release of bacterial MVs. Results demonstrated that neither the release of bacterial nor host cell vesicles was altered by any of the drugs. Moreover, except for Mrc, whose growth was significantly reduced by AZI, bacterial growth was not affected by any of the drugs. In contrast, the release of TNF-α by vesicle-stimulated macrophages was significantly reduced by the glucocorticoids, but not by AZI.

Recently it has been shown that non-typeable *Haemophilus influenzae* exposure to the glucocorticoid beclomethasone results in a phenotype that resembles the *RpoE* knock-out phenotype, and is characterized by an enhanced antibiotic resistance and biofilm formation (Earl et al. 2015). The activation of this *RpoE* gene, which is part of the *σ* ^E^ stress response pathway, has been associated with the release of MVs by Gram-negative bacteria (Schwechheimer et al. 2014). This inspired us to hypothesize that glucocorticoids might inhibit the release of bacterial MVs. However, we found that neither FLUT nor BUD at concentrations that can be attained locally on inhalation affected the release of bacterial MVs.

Moreover, several antibiotics have also been shown to induce the release of bacterial MVs (Hakenbeck et al. 1983; Kadurugamuwa and Beveridge 1995; Schaar et al. 2011b). In our hands, however, AZI at a concentration that is achieved locally in vivo on treatment did not affect the MV release, neither during infection nor in culture. Remarkably, only the bacterial growth of Mrc was significantly reduced by AZI. We also anticipated that the growth of NTHi and Spn would have been inhibited since the minimum inhibitory concentrations (MICs), which were determined for these bacteria using azithromycin sensitivity testing strips, are far below the AZI concentration used in our experiments: NTHi: 0.125; Mrc: 0.125, Spn: 1.5 µg/mL. For Psa, we obtained a MIC of 12 µg/ml which may explain why no reduced bacterial numbers were observed on AZI treatment of Psa. These results were also consistent with previously reported MIC values for NTHi (0.25–4 µg/ml), Mrc (0.25–0.5 µg/ml), and 8–512 µg/ml for Psa but not for Spn (0.25–0.5 µg/ml) (Leclercq et al. 2013; Imperi et al. 2014). The reason for this discrepancy remains unclear as it has been shown that azithromycin can be bactericidal already at early time points (Drago et al. 2005). As it has been shown previously that MVs can contribute to the resistance against certain antibiotics (Schwechheimer and Kuehn 2015), it will be interesting to determine whether MVs participate in the increased azithromycin resistance.

As these drugs are known for their immunosuppressive properties, we additionally determined their effects on the release of TNF-α by naïve macrophages in response to bacterial MVs. We found that both budesonide and fluticasone inhibited the membrane vesicle-induced TNF-α release. This was in line with previous studies that demonstrated that BUD and FLUT have anti-inflammatory properties (Khair et al. 1994; Fattal-German et al. 1996; Ek et al. 1999; Brusselle and Joos 2014). Although some studies have acknowledged an anti-inflammatory effect to AZI (Čulić et al. 2001; Brusselle and Joos 2014), we did not observe a reduced pro-inflammatory response to bacterial MVs after AZI treatment.

It should be noted that in our studies we used a THP-1 macrophage cell model. These PMA-differentiated THP-1 cells show a high similarity with monocyte-derived macrophages both in morphology and behavior (Daigneault et al. 2010). Yet, it cannot be excluded that their responsiveness regarding MV release following infection might be different from alveolar macrophages whose phenotype might be slightly different because of differences in the local micro-environment (Hussell and Bell 2014). Several studies indicate that the release of MVs in response to an inflammatory stimulus (i.e., LPS) occurs in a similar fashion by THP-1 and by alveolar macrophages (Eltom et al. 2014; Soni et al. 2016); however, alveolar macrophages should be used in future studies before definite conclusions on the vesicle release in the context of infection can be drawn. Moreover, we did not determine the effects of BUD, FLUT, and AZI on the release of vesicles under more physiologically relevant conditions, i.e., in the presence of pro-inflammatory mediators or during cigarette smoke exposure. It cannot be excluded that these conditions affect the sensitivity to treatment. Finally, this study only quantified the MVs on treatment with BUD, FLUT, or AZI, but did not characterize the MVs. Vesicle characterization may reveal whether these agents are able to affect the composition and thereby possibly the physiological aspects of these vesicles. Therefore, this may be a subject for further investigation.

To conclude, glucocorticoids and antibiotics are frequently prescribed to treat COPD patients in particular during an exacerbation (Walters et al. 2009; Laue et al. 2015; Ram et al. 2016). Treatment with glucocorticoids is considered an effective treatment for COPD patients that experience an exacerbation, as it increases the rate of lung function improvement and treatment success mostly because it significantly reduces local inflammation (Walters et al. 2009; Jen et al. 2012). Regarding the use of antibiotics, up to 50% of all exacerbations are associated with bacterial infections (Wilkinson et al. 2006; Hurst et al. 2006; Papi et al. 2006; Bafadhel et al. 2011). Therefore, this study aimed to determine how these drugs affect several aspects that to some extent may determine the course of infection: the bacterial growth, vesicle release, and pro-inflammatory response to these MVs. Our study shows that the bacterial growth and MVs release are not affected by FLUT, BUD, or AZI treatment. Treatment with FLUT and BUD, however, was efficient to reduce the pro-inflammatory response to bacterial MVs. The implications of these findings with respect to the effects of these glucocorticoids on airway inflammation and on the resolution of infection need to be further investigated.
